# Timing of Anticoagulation Resumption in a Patient With Mechanical Heart Valves and Spontaneous Subdural Hematomas: A Case Report

**DOI:** 10.7759/cureus.87043

**Published:** 2025-06-30

**Authors:** Sheon Baby, Lucian Lozonschi

**Affiliations:** 1 Internal Medicine, University of Florida, Gainesville, USA; 2 Cardiothoracic Surgery, University of South Florida, Tampa, USA

**Keywords:** mechanical valves, spontaneous intracranial hemorrhage, subdural hematomas, systemic anticoagulation, thromboembolic disease

## Abstract

Managing anticoagulation in patients with mechanical heart valves who develop a spontaneous intracranial hemorrhage is a challenging clinical scenario. Currently, there are no established guidelines on the optimal timing for the resumption of anticoagulation in this high-risk population. We report a case of a 61-year-old male with mechanical mitral and aortic valves on warfarin therapy for 9 years who presented with atraumatic subdural hematomas. The patient’s anticoagulation was reversed, and he underwent middle meningeal artery embolization. Early resumption of anticoagulation resulted in worsening subdural hematomas requiring craniotomies. Holding anticoagulation for 14 days and reinitiating with warfarin alone, without a heparin bridge, prevented subsequent intracranial hemorrhage and preserved valve function. In patients with mechanical heart valves who develop spontaneous subdural hematomas, a strategy of holding anticoagulation for 14 days, followed by reinitiation with warfarin alone, without a heparin bridge, may be a viable approach to prevent recurrent intracranial hemorrhage.

## Introduction

Patients with mechanical heart valves (MHVs) require lifelong anticoagulation with warfarin to prevent thrombotic complications such as valve thrombosis or systemic thromboembolism [1]. However, a challenging clinical scenario emerges when patients with MHVs present with spontaneous intracranial hemorrhage (ICH). In the acute phase of an ICH, anticoagulation is reversed with prothrombin complex concentrate to prevent expansion of the hematoma [2]. This inherently increases the risk for thromboembolic events. There are currently no definitive guidelines regarding the safety of withholding anticoagulation in patients with MHV and ICH, and there are limited data with a wide range of 3 to 21 days [3]. Therefore, the optimal window to resume anticoagulation in this special patient population remains uncertain. We present a case of a patient with atraumatic subdural hematomas while on therapeutic anticoagulation for his mechanical mitral and aortic valves.

## Case presentation

**Figure 1:**
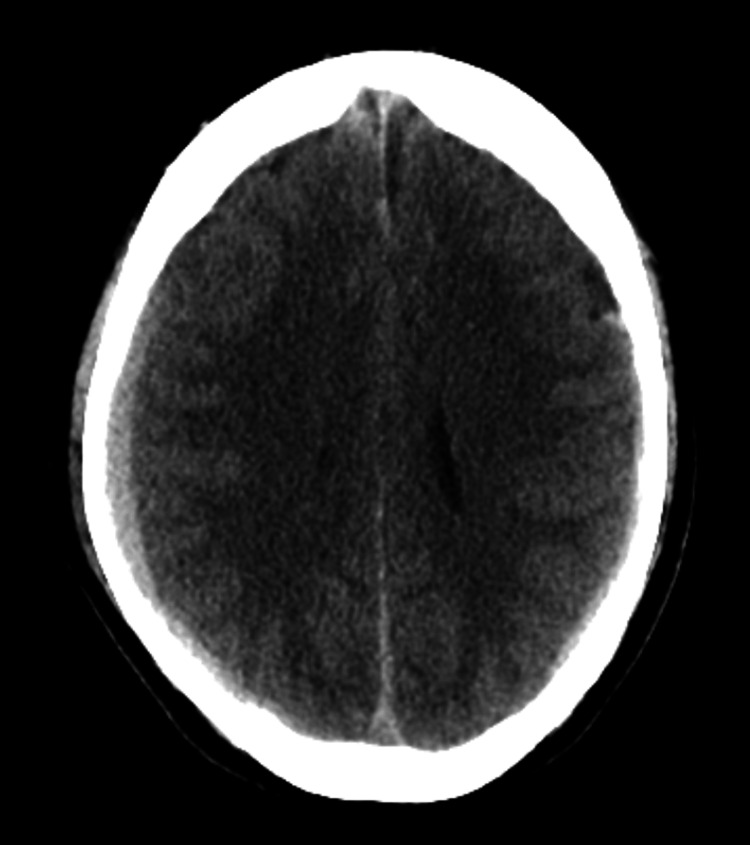
CT head at initial presentation displaying 9 mm right and 3 mm left acute-on-chronic subdural hematomas, with a 1 mm leftward midline shift

A 61-year-old Indian male with a history of migraines and rheumatic heart disease, who had undergone mitral and aortic mechanical valve replacements, presented with a one-week history of mild right-sided headache. The patient had been on warfarin therapy for nine years to prevent thromboembolic complications due to his mechanical heart valves. A CT scan of the head revealed a 9 mm right and 3 mm left acute-on-chronic subdural hematomas, with a 1 mm leftward midline shift (Figure 1). The patient denied any trauma and had no previous history of bleeding complications from warfarin therapy. His international normalized ratio (INR) was therapeutic at 2.6 (goal INR 2.5-3.5), and anticoagulation was reversed.

**Figure 2:**
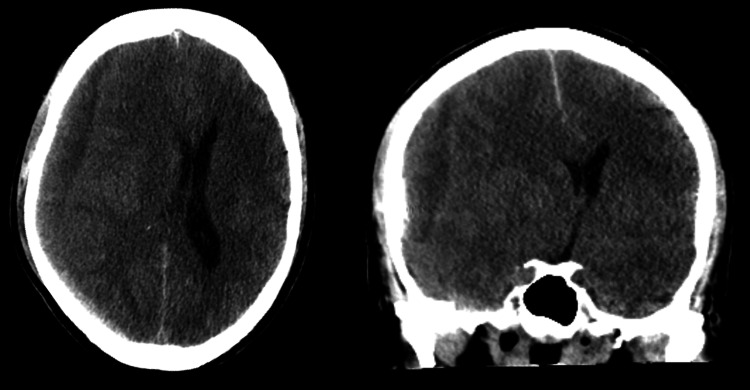
CT head axial (left) and coronal views (right) displaying intracranial hemorrhage expansion: 17 mm right subdural hematoma and 11 mm leftward midline shift with uncal herniation

Given the patient’s need for lifelong anticoagulation and the risk of worsening subdural hematomas, bilateral middle meningeal artery embolization was performed on hospital day 3. Anticoagulation with warfarin and a heparin bridge was resumed the next day. However, 16 days post-reinitiation, the patient experienced worsening headaches, generalized weakness, and an episode of projectile hematemesis. Repeat CT imaging revealed worsening subdural hematomas with significant midline shift and uncal herniation (Figure 2). The patient underwent emergent surgical evacuation with a right parietal craniotomy. Anticoagulation was restarted with a heparin bridge on postoperative day 3. Unfortunately, 13 days later, the patient required a repeat craniotomy due to worsening subdural hematoma.

Given the high risk of recurrent intracranial bleeding, anticoagulation was held for 14 days and then reinitiated with warfarin alone, without a heparin bridge. No further episodes of intracranial hemorrhage occurred. At the one-year follow-up, the patient had no reported thromboembolic events and had normally functioning mechanical valves on a transthoracic echocardiogram.

## Discussion

Our patient did not benefit from middle meningeal artery embolization to prevent recurrent intracranial hemorrhage. Furthermore, trials of early resumption of therapeutic anticoagulation resulted in an expansion of the subdural hemorrhage, which was clinically significant within two weeks. A recent retrospective study evaluated 184 patients with MHVs and ICH. They found that the mean time from ICH to anticoagulation was 12.7 days [1]. Furthermore, they found no difference in 30-day thrombotic and hemorrhagic brain-related outcomes when anticoagulation was resumed early (within 7 days) versus late (within 7 to 30 days) after ICH. Yet another retrospective study examining 63 patients concluded that holding anticoagulation for at least 10 days significantly reduced hemorrhagic events while having no significant difference on thromboembolic events [3]. A third European study with 137 patients found that restarting therapeutic anticoagulation within less than two weeks after ICH in patients with MHVs was associated with increased hemorrhagic complications. Furthermore, the earliest time to restart anticoagulation was six days for patients with a high thromboembolic risk [2].

Overall, the current literature seems to favor holding anticoagulation for the first one to two weeks after an intracranial hemorrhage in patients with MHVs [2-6]. Perhaps, the small differences in conclusions between these studies are due to the diverse patient population that is sampled. The timing of anticoagulation resumption should be personalized to each patient. Patients with high-risk features, such as the need for hematoma evacuation or a midline brain shift, as in our patient, should have anticoagulation held for a longer time than those without these significant risk factors. Other risks to consider include the location of the prosthetic valve, as a mechanical mitral valve has a higher thromboembolic risk than a mechanical aortic valve [3,7]. Patients with a history of valve thrombosis, atrial fibrillation, or deep vein thrombosis have a higher hypercoagulable risk profile and may warrant earlier initiation of anticoagulation [3]. Lastly, the type of intracranial hemorrhage is important to distinguish. It is possible that patients with a reversible etiology, such as traumatic ICH, may be able to reinitiate anticoagulation sooner, once the underlying trauma is resolved, than patients without a reversible etiology such as spontaneous ICH. More studies will need to be conducted to further investigate this possibility.

The latest retrospective study evaluating 171 patients concluded that holding anticoagulation for at least seven days may be safe in patients with MHVs [6]. Additionally, heparin bridging was associated with a higher risk of bleeding than warfarin alone [6]. Similarly, our patient demonstrated that holding anticoagulation for 14 days was safe, and bridging with a heparin drip was not required. However, our patient did not have other risk factors for thrombosis such as a previous history of stroke or known valvular thrombosis. Additionally, our patient had risk factors for subsequent hemorrhage, including a midline shift on CT scan and spontaneous ICH without a reversible etiology. Thus, it is important to consider the individual risk-benefit profile to determine the timing of anticoagulation reinitiation.

## Conclusions

Our case report suggests that holding anticoagulation for 14 days and restarting anticoagulation with only warfarin, without a heparin bridge, may be a safe option in spontaneous subdural hematomas. However, larger studies will be needed to further elucidate the optimal timing of anticoagulation resumption in this special patient population.
